# Surface Stabilization of High-Pressure TiO_2_ Polymorph via High-Energy Ball Milling: Boosting Noble-Metal-Free CO_2_ Photoreduction

**DOI:** 10.1021/acsomega.5c10398

**Published:** 2026-03-13

**Authors:** Abigail Mufari, Thiago Capelupi, Martin Saleta, Eugenia Zelaya, Octavio Furlong, Maria Valnice Boldrin Zanoni, Luis Eduardo Cadús, Juliana Ferreira de Brito, Sebastián Larrégola

**Affiliations:** † Instituto de Investigaciones en Tecnología Química (INTEQUI), UNSL—CONICET, Almirante Brown 1455, 5700 San Luis, Argentina; ‡ Universidade Estadual Paulista (UNESP), Instituto de Química, Araraquara 14800-060, Brazil; § Instituto de Nanociencia y Nanotecnología (INN), CNEA-CONICET, Centro Atómico Bariloche, Av. Bustillo 9500, 8400 S. C. de Bariloche, RN, Argentina; ∥ Instituto Balseiro, Universidad Nacional de Cuyo and CNEA, Av. Bustillo 9500, 8400 S. C. de Bariloche, RN, Argentina; ⊥ Centro Atómico Bariloche, Comisión Nacional de Energía Atómica, Av. Bustillo 9500, 8400 S. C. de Bariloche, RN, Argentina; # Instituto de Física Aplicada, (INFAP), UNSL-CONICET, Av. Ejército de Los Andes 950, 5700 San Luis, CP, Argentina

## Abstract

High-energy ball milling (HEBM) is employed to stabilize the high-pressure TiO_2_–II polymorph as nanocrystallites anchored to anatase surfaces, producing a controllable polymorphic mixture that markedly enhances CO_2_ photoreduction to CH_3_OH in aqueous media without noble metals or cocatalysts. The resulting architecture features TiO_2_–II intimately interfaced with strained anatase and a high density of extended defects (grain boundaries, phase interfaces, and dislocation terminations) hosting reactive surface species with modified electronic properties. This defect-rich configuration provides high-affinity CO_2_ adsorption and activation sites. Both bulk and surface are profoundly restructured under the extreme nonequilibrium conditions of HEBM, which reproduce high-pressure transformation pathways at ambient conditions. These results highlight a green, scalable strategy for defect and polymorph engineering in TiO_2_, enabling targeted surface chemistry design to improve photocatalytic CO_2_ conversion.

## Graphical abstract

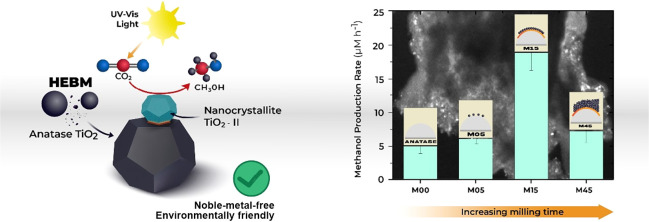

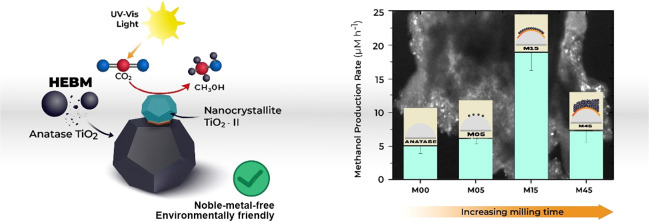

## 1 Introduction

Photoreduction of CO_2_ is emerging as a critical pathway in the search for solar fuels, with the potential to transform carbon dioxide into valuable energy carriers. This process mimics natural photosynthesis, using UV–vis radiation to drive the chemical reduction of CO_2_, offering a sustainable route for fuel production that could significantly reduce dependence on fossil resources.^1–4^ The efficiency of CO_2_ photoreduction remains a major challenge, mainly due to the complex multielectron transfer processes and the high stability of the reactant molecule.^5^ Current photocatalytic materials often suffer from low quantum efficiency, limited light absorption, and rapid charge recombination, restraining their practical applications.^6,7^ Hence, the development of advanced photocatalysts that significantly improve the efficiency of the photoreduction process is essential to establish solar fuel production as a practical and sustainable energy solution for the future.

TiO_2_ anatase is one of the most effective and used photocatalytic materials due to its favorable optical and electronic properties. Its wide bandgap (∼3.2 eV) allows it to harvest ultraviolet light,^8^ plus its high stability, nontoxicity, and strong oxidizing power make it an attractive candidate for photooxidation applications.^9^ Despite these advantages, it presents several limitations: The wide bandgap restricts the utilization of visible light, which comprises the majority of the solar spectrum, thereby limiting the material’s effectiveness in natural sunlight. The rapid recombination of photogenerated charge carriers further diminishes its photocatalytic performance, with recombination rates that can reach up to 90% within nanoseconds.^6,7^ These challenges highlight the need for innovative strategies to modify or enhance TiO_2_ anatase properties to improve its performance, such as doping with metals, noble metals, or nonmetal elements, self-doping procedures, heterojunctions, etc.^10–16^

Within these strategies, high-pressure torsion experiments (HPT) have shown a positive impact on the structure and activity of TiO_2_ anatase toward H_2_ photogeneration under visible light.^17^ HPT treatments were applied to TiO_2_-Anatase, stabilizing a high-pressure TiO_2_–II polymorph at the surface with large fractions of crystal defects by inducing plastic strain to anatase under 6 GPa and room temperature. The H_2_ photoproduction increases 5 times under visible light irradiation, associated with a TiO_2_–II weight fraction increase to 60%. The removal of oxygen vacancies from the material by annealing increases the performance of the photocatalytic H_2_ generation.

High-energy ball milling (HEBM) is a powerful and green method that can be used to modify the structural and surface properties of materials. This technique induces significant mechanical forces that can alter crystal structure, reduce particle and/or crystallite size, and affect surface and bulk composition. In the case of TiO_2_ anatase, depending on the milling parameters (rotation speed, mass ratio, grinding media material, grinding time, etc.), HEBM can lead to a series of structural transformations, following the path: Anatase → TiO_2_–II → Rutile. These structural modifications are crucial, as they can significantly influence the photocatalytic properties of the material.^18,19^

Titanium dioxide (TiO_2_) naturally exists in three primary polymorphic forms: anatase, brookite, and rutile, with rutile being the thermodynamically stable phase under equilibrium conditions and several metastable structures.^20^ TiO_2_–II presents a compacted orthorrombic α-PbO_2_-type crystal structure and it has been stabilized through static high-pressure/high-temperature treatments, HPT experiments^17^ and HEBM.^21–27^

Kinetic studies of the phase transformation from anatase to TiO_2_–II and rutile during HEBM reveal influences of the milling parameters and the initial particle size. The formation of TiO_2_–II has been proposed to occur within the powder volume trapped during collisions. Microstructural analysis shows that for specific milling times, the particle size and specific surface areas remain largely unchanged, suggesting that the transformation from nanocrystalline anatase to TiO_2_–II can occur without the typical breaking and welding processes associated with mechanical alloying.^26^ The transformation is notably influenced by the initial size of the anatase particles; smaller particles tend to undergo a more complete transformation to TiO_2_–II, whereas bigger particles primarily form TiO_2_–II at the particle surface, leading to superficial roughness attributed to TiO_2_–II nanograins.^28,29^ Milling can reduce the crystallite size of anatase from hundreds of nanometers down to sub-10 nm, increasing the specific surface area and enhancing the material’s ability to adsorb reactant molecules.^25^

The surface properties of milled anatase nanopowders indicate that the density of surface reactive sites, particularly hydroxyl (OH) groups, increases with milling time, implying enhanced surface reactivity.^30^ Despite the increase in active surface group concentration, a decline in photocatalytic activity has been reported for grinding at mild and intense conditions. It has been proposed that the introduction of new photoluminescence (PL) states with high quantum efficiency generates additional recombination channels, which reduce the number of electron–hole pairs available for photocatalysis, particularly for the ^•^OH radical generation, negatively affecting photo-oxidation of diverse compounds: NO, salicylic acid, propane, etc.^30–32^ Photoreduction of Cr­(VI) is affected in the same way in grinded anatase. This has been related to a decrease in the anatase concentration at the surface and an increase in the concentration of surface recombination centers, reducing in this way its availability for the photocatalytic process.^33^

This study focuses on the production of TiO_2_-based nanocomposites with diverse polymorphs via high-energy ball milling. We investigated their surface and bulk properties and their photocatalytic performance in methyl orange (MO) degradation and CO_2_ photoreduction, with a particular emphasis on HEBM-stabilized TiO_2_-Anatase/TiO_2_–II nanocrystalline systems. This investigation aims to contribute to the development of more effective, noble-metal-free strategies for photocatalytic CO_2_ reduction by elucidating the impact of HEBM-induced surface and bulk modifications, thereby addressing critical environmental challenges.

## 2 Experimental Section

### 2.1 Synthesis of Materials

The materials were synthesized using HEBM in a Fritsch Pulverisette 6 planetary ball mill, equipped with an 80 mL vial and tungsten carbide balls (*d*_B_ = 15 mm). The solid-to-ball mass ratio was maintained at 1:35 and the starting material was commercial TiO_2_-anatase. The milling process was performed at 350 rpm for durations of 5, 15, and 45 min in air atmosphere. Following milling, the samples were calcined at 450 °C for 2 h. The resulting samples are denoted as M*X*, where *X* indicates the milling time in minutes: 0 (M00), 5 (M05), 15 (M15), and 45 (M45).

### 2.2 Bulk Characterization: Structure, Morphology, and Electronic Defect Analysis

Crystal structure, phase composition, crystallite sizes and unit cell parameters were investigated from X-ray powder diffraction (XRPD). The patterns were collected using a Rigaku Ultima IV diffractometer operating at 20 mA and 30 kV, with Cu Kα radiation (λ = 1.5418 Å), over a 2θ range of 10° to 100°, in step-scan mode with a step size of 0.02° and a counting time of 5 s per step. A silicon standard was used to estimate the average apparent crystallite size (*D*_CRIST_) and the average maximum microstrain (ε_MAX_). The diffraction patterns were further examined through Rietveld refinement^34^ using the Fullprof software package.^35^

The morphology was characterized by TEM. These studies were carried out using a CM 200 Philips microscope, equipped with an ultratwin objective lens and operated at an acceleration voltage of 200 kV (LaB_6_ filament). We studied three samples (M00, M15, and M45). Powder of each sample was dispersed in isopropyl alcohol, employing an ultrasonic bath to obtain a colloidal solution. Drops of this colloid were deposited over commercial Formvar coated Cu grids. Dark-field (DF) TEM images were acquired in transmission mode. DF imaging was performed by selecting a specific diffracted spot in SAED mode, positioning an objective aperture over this reflection, and subsequently switching to image mode. Under these conditions, bright contrast arises exclusively from crystallites contributing to the selected diffraction spot, enabling phase-selective imaging of nanodomains belonging to crystallographically distinct phases relative to the anatase matrix.

The magnetic characterization of the system was performed with EPR measurements. The EPR spectra were acquired in a Bruker ELEXSYS II-E500 spectrometer (X-band—9.5 GHz). The paramagnetic signal was collected with an attenuation of 25 dB (0.6 mW microwave power) and a modulation field of 2 Oe amplitude. The powder sample was measured in an ESR quality quartz tube at 114 K, performing 10 scans to improve the signal-to-noise ratios.

### 2.3 Photocatalysis: MO Photodegradation and CO_2_ Photoreduction

CO_2_ photoreduction was carried out in a sealed, hermetic, single-chamber reactor equipped with a quartz window, 100 mL of ultrapure Milli-Q water saturated by CO_2_, and 100 mg of M*X*. The reduction reaction was carried out under stirring and under UV–vis light (300W xenon lamp, Oriel, ∼600 mW cm^–2^) for 1 h. The spectrum of Xe light measured with Ocean Optics USB4000 Fiber Optic Spectrometer is presented in Figure S.1. The products were quantified after a solid-phase microextraction technique via gas chromatography using a Shimadzu GC-2010 Pro with a flame ionization detector (GC-FID, Shimadzu), equipped with a 30 m × 0.25 mm Stabilwax column.^36^ The run started at 57 °C for 4 min, followed by a heating ramp of 45 °C min^–1^ until 170 °C, maintained for 4 min. The injector and the FID detector were set at 240 and 260 °C, respectively, and the chosen carrier gas was N_2_, with a total flow of 14 mL min^–1^.^37^

The MO photodegradation experiments were conducted in a Pyrex photoreactor equipped with a cooling jacket at 15 °C. The solar simulator employed in these experiments was an Xe arc lamp (ORIEL, LSC-100), positioned 5 cm from the reactor. In each reaction test, 100 mg of the catalyst was dispersed in 100 mL of an HCl/KCl buffer solution (pH = 2) containing a dye concentration of 10 mg L^–1^. The mixture was magnetically stirred in darkness for 30 min before irradiation to allow the catalyst to absorb the dye and ensure uniform dispersion. Throughout the illumination process, the photocatalytic system was kept under magnetic agitation. After a 1 h reaction period, 5 mL aliquots were collected and analyzed using a Cary 60 UV–vis spectrophotometer. The maximum absorbance value represents the highest concentration of MO.

### 2.4 Surface Chemistry: Composition and Physical Properties

Specific surface area was determined using the BET method through nitrogen adsorption measurements at 77 K. Before analysis, samples were degassed at 200 °C to ensure the removal of adsorbed moisture and surface impurities. A Micromeritics Gemini instrument, for high-precision surface area measurements, was used to conduct the adsorption experiments. The specific surface area was calculated by applying the BET equation to the nitrogen adsorption isotherms obtained from the measurements.

UV–vis diffuse reflectance spectroscopy (DRS) measurements were performed using a Cary 500 UV–vis spectrophotometer equipped with an integrating sphere, covering the absorption range of 350–700 nm, with MgO used as the reference standard.

X-ray photoelectron spectroscopy (XPS) spectra were collected with a ProvenX-PS system (SPECS) Multitechnique instrument equipped with a Mg/Al dual X-ray source and a PHOIBOS 150 hemispheric analyzer. A pass energy of 10 eV and an Mg anode operated at 300 W was used. The pressure was kept under 1 × 10^–9^ mbar during all measurements. Spectra were obtained for the C 1s, O 1s, and Ti 2p signals as well as for the valence band region of the samples. Adventitious C 1s binding energy (285 eV) was used as internal reference to correct peak positions.

Temperature-programmed desorption of CO_2_ (CO_2_-TPD) was carried out employing a homemade desorption facility with a thermal conductivity detector. 500 mg of sample were placed in the reactor and subjected to surface cleaning using He at 150 °C for 30 min. Adsorption was conducted under a mixed gas flow of CO_2_ and He (20 mL min^–1^ CO_2_ and 70 mL min^–1^ He) for 30 min. After a purge stage in He, the desorption was performed under He flow (30 mL min^–1^) from room temperature to 700 °C at 5 °C min^–1^. The maximum temperature was maintained for 30 min to ensure the completion of all desorption processes.

## 3 Results and Discussion

### 3.1 Bulk Characterization

High-energy ball milling (HEBM) induces severe plastic deformation in TiO_2_, producing a rapid decrease in anatase crystallite size together with the nucleation of the high-pressure TiO_2_–II polymorph. Rietveld refinements (Figure 1a and Table 1) show that pristine anatase (M00) crystallizes in the tetragonal *I*4_1_/*amd* structure (*V* = 136.271(5) Å^3^) and preserves its symmetry upon milling, while its crystallite size decreases from 200.2 nm (M00) to 132 nm (M05), 52.2 nm (M15), and 49.9 nm (M45). The average microstrain (ε_MAX_) increases moderately up to M15 (0.05%) and then sharply in M45 (0.35%), indicating a transition from mild to severe lattice distortion once grain refinement saturates.

**Figure 1:**
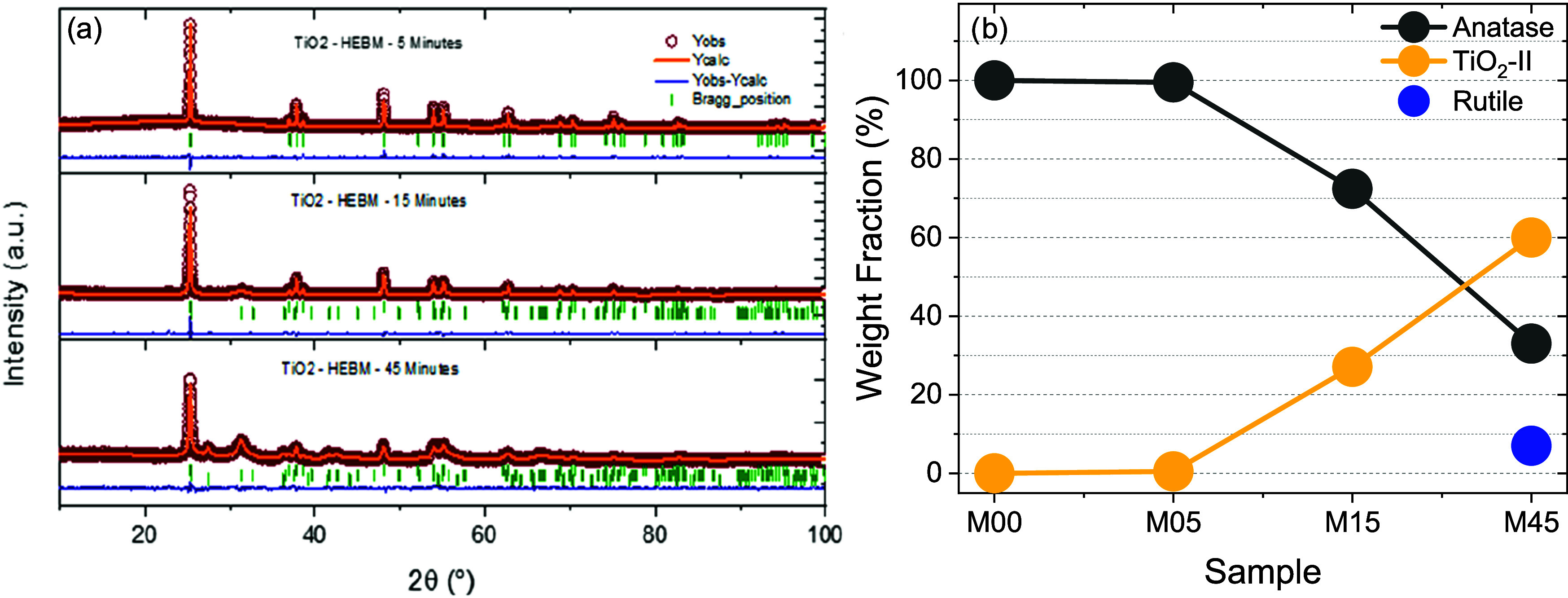
(a) Rietveld refinement plots obtained for M05, M15 and M45 samples. First row of vertical ticks corresponds to Bragg reflections for anatase polymorph, second row to TiO_2_–II and third row of ticks to rutile. (b) Evolution of the polymorphic weight fractions for samples.

**Table 1:** Rietveld Refinement Results, Phase Composition (wt %), Average Crystallite Size (nm), and Unit Cell Parameters (Å, Å^3^) for Samples Obtained from XRPD Data^a^

parameter	M00	M05	M15	M45
polymorph	anatase			
*a* (Å) = *b* (Å)	3.7845(1)	3.7844(1)	3.7852(1)	3.7848(4)
*c* (Å)	9.5143(1)	9.5100(2)	9.5119(3)	9.517(1)
vol. (Å^3^)	**136**.**271(5)**	**136**.**20(1)**	**136**.**29(1)**	**136**.**33(3)**
phase fraction (% wt)	100	>99	72(1)	33(1)
average apparent crystallite size (*D*_CRIST_) [nm]	200.2	132	52.2	49.9
size anisotropy [nm]	0.02	0.07	0.03	0.01
average maximum microstrain (ε_MAX_) [%]	-	0.036	0.055	0.346
microstrain anisotropy [%]	-	<0.0003	<0.0003	<0.0003
*R*_BRAGG_		9.3	5.1	6.0

parameter	M00	M05	M15	M45
polymorph	TiO_2_-II			
*a* (Å)	-	-	4.555(5)	4.553(2)
*b* (Å)	-	-	5.469(5)	5.485(3)
*c* (Å)	-	-	4.928(4)	4.920(2)
vol. (Å^3^)	-	-	**122.8(2)**	**122.9(1)**
phase fraction (% wt)	-	<1	28(1)	60(1)
average apparent crystallite size (*D*_CRIST_) [nm]	-	-	5.9	6.4
size anisotropy [nm]	-	-	0.02	0.005
average maximum microstrain (ε_MAX_) [%]	-	-	0.298	0.486
microstrain anisotropy [%]	-	-	<0.0003	0.00059
*R*_BRAGG_	-	-	10.2	3.9

a Data from rutile (7%) at the M45 sample, are not shown in this Table.

New reflections at 2θ = 31.6°, 41.8°, and 43.1° correspond to TiO_2_–II (see Figure S.2), whose refined unit cell volume in M15 and M45 (122.8(2) Å^3^ and 122.9(1) Å^3^) is slightly larger than the value reported for stoichiometric TiO_2_–II synthesized under HP/HT conditions (121.95 Å^3^).^38^ TiO_2_–II appears at <1 wt % in M05, increases to 28(1)% in M15, and reaches 60(1)% in M45, where rutile (7%) also emerges (Figure 1b). After 360 min of milling a single rutile phase is stabilized as the thermodynamic product of the procedure (Figure S.3). The crystallite size of TiO_2_–II remains in the 5–6 nm range and exhibits high microstrain (ε_MAX_ = 0.298–0.486%), consistent with a highly defective framework. These microstructural features, grain-size reduction, high strain, and phase transformation, are characteristic of intense HEBM-induced deformation.^28,30^

TEM analyses support the XRPD results. M00 and M15 exhibit submicrometric grains with ⟨*d*⟩ ≈ 158 nm (log-normal distribution; Figures S.4 and S.5). SAED patterns (Figure 2) confirm the coexistence and progressive dominance of TiO_2_–II and rutile at longer milling times. HRTEM imaging of M15 reveals small regions with interplanar distances compatible with strained TiO_2_–II (Figure S.5), while dark-field images show 20–30 nm surface regions enriched in this polymorph (Figures 3 and S.6). In the dark-field image (Figure 3b), contrast arises exclusively from crystallites contributing to the most intense diffraction ring of TiO_2_–II; in this case, only particles smaller than 50 nm diffract into this ring and therefore appear bright, indicating that TiO_2_–II is preferentially present as sub-50 nm nanodomains at the anatase surface. In M45, a bimodal size distribution emerges: large particles comparable to M00 coexist with smaller particles localized mainly on the surface of anatase (Figures 4 and S.7).

**Figure 2:**
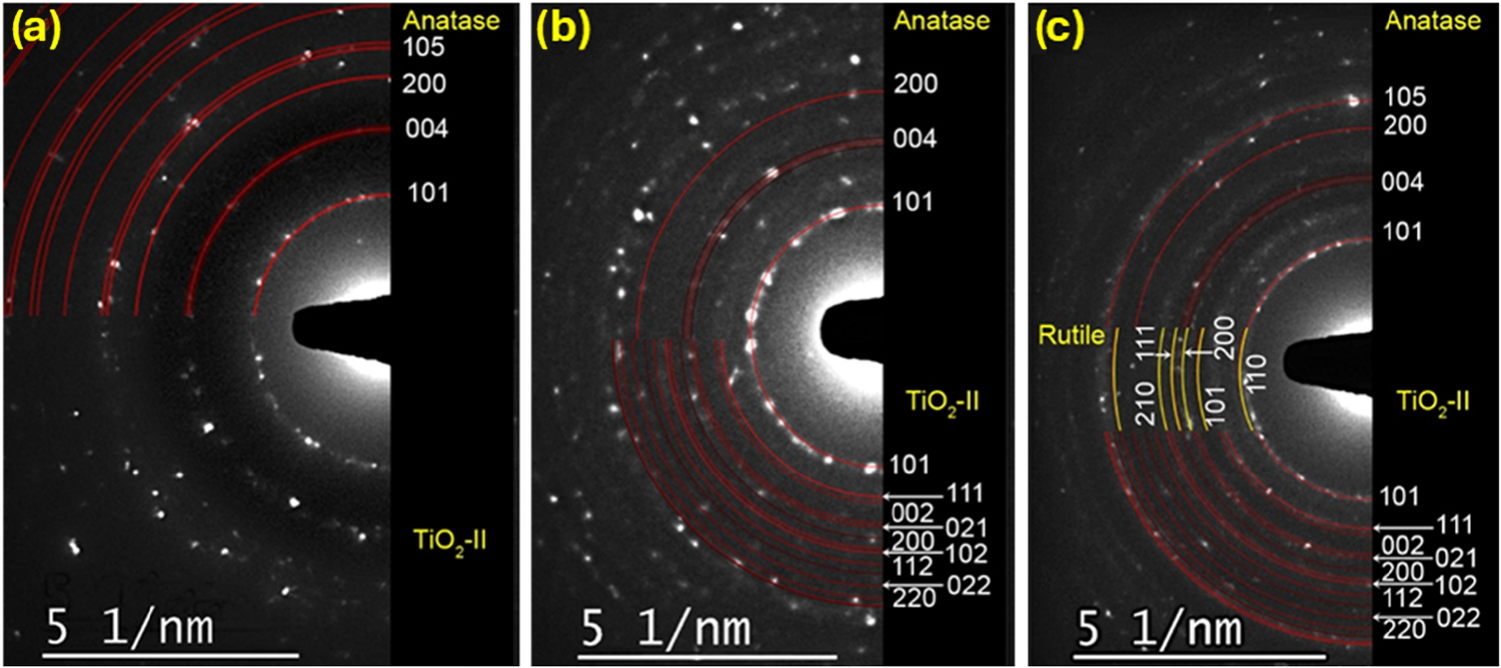
SAED pattern of (a) M00 (left), (b) M15 (middle), and (c) M45 (right) samples.

**Figure 3:**
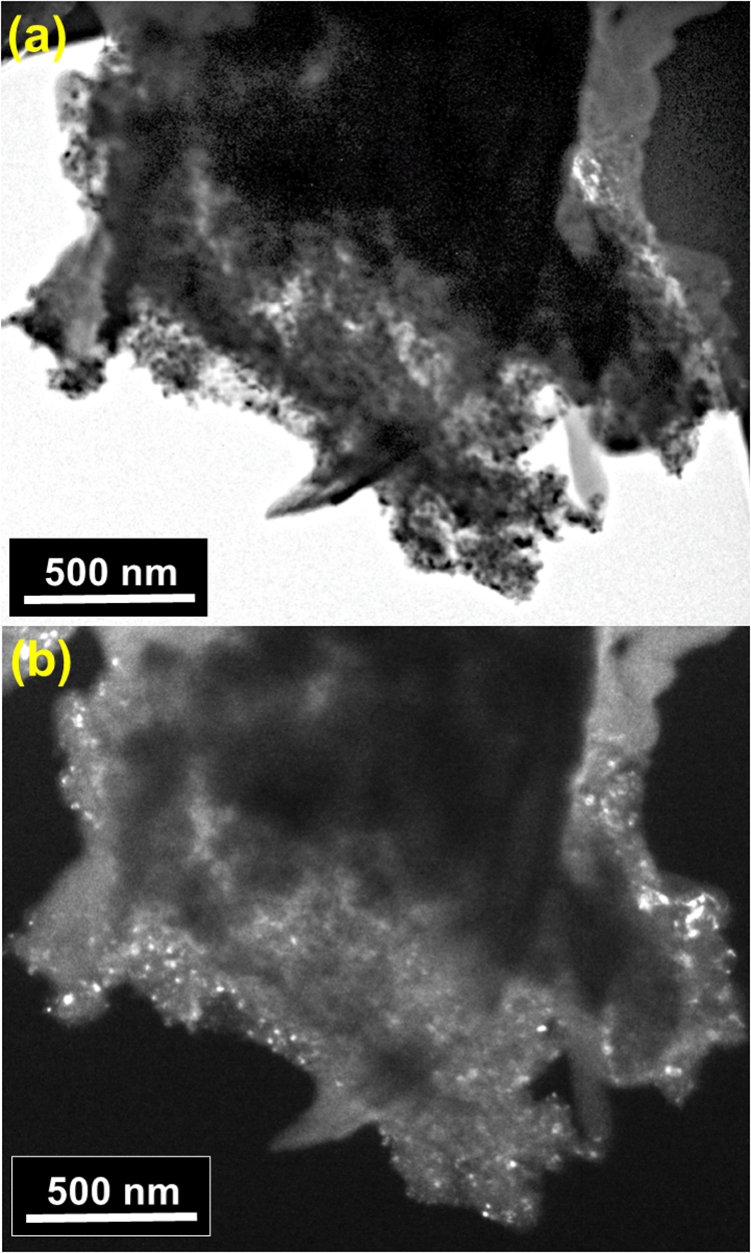
M45 micrographs. (a) Bright field and (b) dark field images. The dark-field images were constructed by positioning the objective aperture at the position of the TiO_2_–II diffraction rings. In these images, the presence of particles with a size smaller than 50 nm is evident.

**Figure 4:**
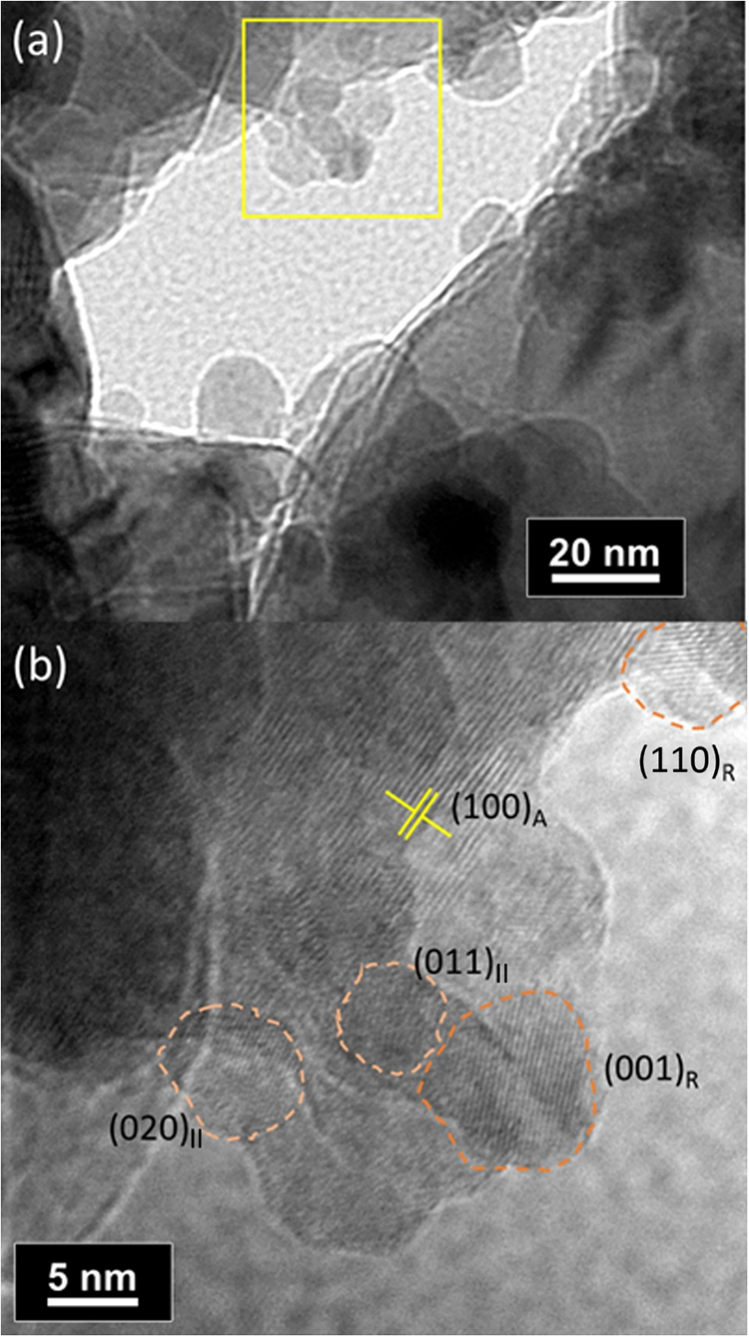
TEM images of the M-45 sample. (a) High-magnification image. (b) Detail of the marked area in (a). The observed grains are outlined, and their corresponding TiO_2_ phases are identified based on the measured spacing between crystallographic planes. Additional grains are present but not highlighted due to insufficient contrast, which hinders the measurement of interplanar distances. Average distances presented: (020)_II_ = 0.27(2) nm; (011)_II_ = 0.35(1) nm, (001)_R_ = 0.30(1) nm; (110)_R_ = 0.32(1) nm and (100)_A_ = 0.39(1) nm.

This polymorphic configuration produces bulk and surface regions enriched in extended defects, anatase grain boundaries, TiO_2_–II grain boundaries, anatase/TiO_2_–II interfaces, and dislocation terminations, potential reactive undercoordinated sites with modified electronic properties. DFT studies of TiO_2_ heterointerfaces highlight interface-localized charge trapping behavior,^39^ and experimental observations show that electrons and holes preferentially localize at undercoordinated surface sites in anatase.^40^

The increase in *S*_BET_ (Table 2) with milling is consistent with anatase crystallite-size reduction and the presence of TiO_2_–II nanocrystallites. Although higher surface area generally favors adsorption, the strong strain developed in both anatase and TiO_2_–II, along with the formation of defective heterointerfaces, is expected to play a more relevant role in the following surface-dependent processes.

**Table 2:** Estimated Indirect Band Gap Energies (*E*_g_, eV) for M00, M05, M15, and M45 Samples Derived from Tauc Plots of UV-Vis DRS Data^a^

sample	*E*_g_ (eV)-main absorption	*E*_g_ (eV)-second absorption	*S*_BET_ (m^2^/g)	predominant phase
M00	3.20	-	8.1	anatase
M05	3.10	2.98	10.2	anatase
M15	3.16	2.98	11.3	anatase/TiO_2_–II interplay
M45	2.98	-	14.1	TiO_2_–II

a Specific surface area (*S*_BET_).

The paramagnetic behavior was studied through X-band EPR at 114 K, and the corresponding spectra are presented in Figure 5a. Pristine anatase (M00) exhibits five signals of variable intensities at *g* = 2.004(1), 1.991(1), 1.974(1), 1.947(1), and 1.931(1). In TiO_2_ anatase, *g*-values above 2 are commonly attributed to oxygen-related defects, including surface or bulk oxygen vacancies and trapped-hole centers, while *g*-values below 2 are generally associated with Ti^3+^ species or trapped electrons located at distinct lattice or subsurface sites. This assignment is supported by prior studies: Hurum et al.^41^ identified the signal at *g* = 1.991, with a shoulder at *g* = 1.957, as arising from lattice electron-trapping sites; Chiesa et al.^42^ reported anisotropic components at *g*_⊥_ = 1.992 and *g*_∥_ = 1.960, typical of Ti^3+^ in oxygen-deficient environments; Naldoni et al.^43^ assigned *g* = 1.993 and 1.964 to Ti^3+^ in regular lattice sites; and the signals at *g* = 1.972 and 1.947 have been unambiguously linked to bulk Ti^3+^ centers in a distorted rhombic ligand field.^44^ A minor contribution from surface Ti^3+^ is also detected at *g* = 1.93 in M00, consistent with reports on surface-stabilized Ti^3+^ states generated by different synthetic or postsynthetic routes.^41,45^ This surface-related signal is absent in M05, M15, and M45, indicating that HEBM rapidly suppresses surface Ti^3+^ centers.

**Figure 5:**
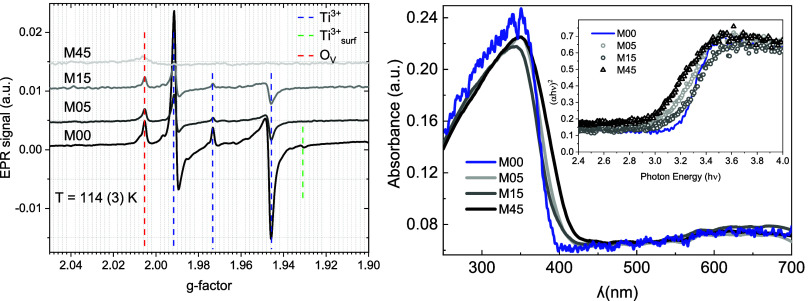
(a) X-band EPR spectra measured at 114 K for the samples. (b) DRS spectra presented as absorbance against wavelength. Inset: Tauc plots for indirect band gap transitions for TiO_2_ samples: M00, M05, M15, and M45.

The signal at *g* = 2.004, close to the free-electron value (*g* = 2.0023), may originate from either oxygen vacancies containing a trapped electron or trapped-hole O^–^ species,^46^ both of which have been widely reported in anatase.^47,48^ Because these states can occur in surface or subsurface regions and are highly sensitive to local structural distortions, their precise microscopic assignment remains under debate.

After HEBM, no new paramagnetic resonances appear; however, significant changes occur in the relative intensities of the existing signals. The *g* = 2.004 feature decreases markedly from M00 to M05 and remains nearly constant thereafter. The surface Ti^3+^ signal at *g* = 1.93 disappears after only 5 min of milling, and no indications of O_2_^–^ formation via O_2_–Ti^3+^ interaction are observed.^49^ When considering the anatase weight fractions obtained from Rietveld refinements, the intensities of the bulk Ti^3+^-related signals (*g* < 2.000) remain approximately constant between M05 and M15, indicating minimal variation in the bulk paramagnetic response of anatase within this milling regime. In contrast, for M45 the *g* = 1.991 signal becomes barely detectable, while the *g* = 2.004 band persists with an intensity similar to that of M05 and M15. No Ti^3+^ features attributable to the TiO_2_–II polymorph were detected, despite its predominance in M45, suggesting that this high-pressure phase either does not stabilize paramagnetic Ti^3+^ states under these conditions or hosts EPR-silent defect configurations.

The evolution of paramagnetic centers under HEBM thus reflects distinct surface and bulk modifications. The rapid loss of the surface Ti^3+^ signature (*g* = 1.93) within the first minutes of milling, as previously observed in related systems,^50,51^ indicates a permanent reconfiguration of the outermost layers of anatase. Bulk Ti^3+^ signals persist into M05 and M15 but diminish significantly in M45 as TiO_2_–II becomes the dominant phase. Meanwhile, the oxygen-related feature at *g* = 2.004 remains throughout the series, reflecting the presence of oxygen-centered paramagnetic species in both anatase and TiO_2_–II. Overall, the EPR results show that HEBM suppresses surface Ti^3+^ states, progressively reduces bulk Ti^3+^ centers, and does not generate new paramagnetic signatures associated with TiO_2_–II, pointing to fundamentally different defect-stabilization behavior in the high-pressure polymorph.

### 3.2 UV–Vis Diffuse Reflectance Spectroscopy (DRS)

The absorption spectra of the milled TiO_2_ samples are presented in Figure 5b. All materials exhibit an intense transition below 440 nm, corresponding to the valence-to-conduction band excitation typical of anatase.^52^ In pristine TiO_2_ (M00), this feature dominates the spectrum, yielding an indirect band gap of 3.20 eV (Table 2). Tauc plots for the indirect transition are shown in the inset of Figure 5b, and corresponding linear extrapolations in Figure S.8.

A broad feature at 600–700 nm is observed for all TiO_2_ samples, including commercial anatase. This signal does not correspond to an intrinsic electronic transition but is instead attributed to wavelength-dependent light scattering and thin-layer interference,^53–55^ effects commonly reported for strongly scattering TiO_2_ powders. Consequently, the hump is interpreted as an optical artifact rather than a material-specific absorption

Upon milling, M05 displays a reduced band gap of 3.10 eV, approximately 0.10 eV lower than the pristine sample. This decrease is consistent with the accumulation of structural defects, such as vacancies, grain boundaries, or dislocations, introduced by HEBM in both bulk and surface regions. In addition, a minor absorption feature appears at 2.98 eV, indicating the emergence of a second transition. In M15, the main band gap slightly increases to 3.16 eV and a second absorption at 2.98 eV, stronger than in M05, is again detected (Figure S.8), suggesting an increasing contribution from the high-pressure polymorph.

The spectral behavior of M45 is distinctly different. Here, the principal transition corresponds to *E*_g_ = 2.98 eV, in line with its larger TiO_2_–II content (60%, from Rietveld refinement and TEM). This lower-energy absorption is consistent with reported indirect band gaps of polycrystalline TiO_2_–II obtained by high torsion pressure synthesis (2.7–2.9 eV).^17,56^ The slightly higher *E*_g_ observed in the HEBM sample may reflect a comparatively higher oxygen stoichiometry, possibly influenced by the simultaneous pressure–temperature conditions imposed during milling (2–4 GPa/250–400 °C) and the subsequent air atmosphere calcination step.

Overall, the indirect band gap exhibits a nonmonotonic evolution with milling time: 3.20 eV (M00), 3.10 eV (M05), 3.16 eV (M15), and 2.98 eV (M45); with intermediate variations falling within the experimental uncertainty (±0.03 eV). The lowest value observed for M45 reflects modifications in surface electronic structure associated with the substantial formation of TiO_2_–II and the increased degree of structural disorder. A summary of these optical properties is provided in Table 2.

### 3.3 X-ray Photoelectron Spectroscopy

The chemical composition and surface electronic structure of the samples were investigated by XPS (detection depth 5–10 nm), so that the spectra predominantly probe the outermost layers, corresponding to only a few unit cells, and are thus sensitive to surface and subsurface modifications. Survey spectra reveal Ti, O and adventitious C as the main elements at the surface of all samples (Figure S.9 and Table S.1). The O_TOT_/Ti ratio indicates an oxygen overstoichiometry that slightly decreases with milling time, from 2.70 (M00) to 2.55 (M05), 2.50 (M15) and 2.46 (M45). To gain deeper insight into the structural and electronic changes, high-resolution Ti 2p and O 1s spectra, as well as the valence band (VB) region, were analyzed (Figures 6, 7, and 8).

**Figure 6:**
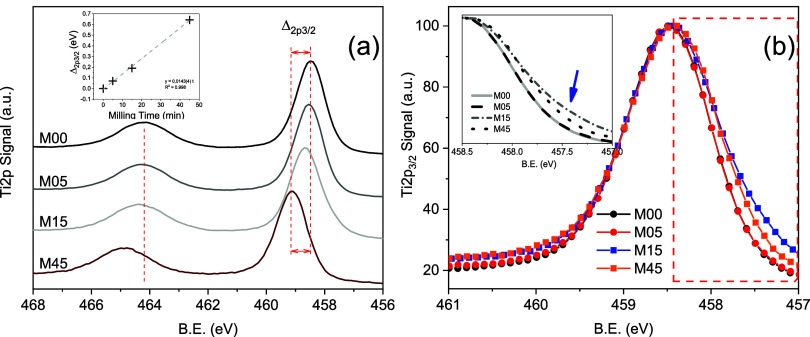
(a) XPS spectra for M00, M05, M15, and M45. Inset: 2p_3/2_ peak binding energy shifting across HEBM. (b) Close up of the normalized Ti 2p_3/2_ peak highlighting low-energy features.

**Figure 7:**
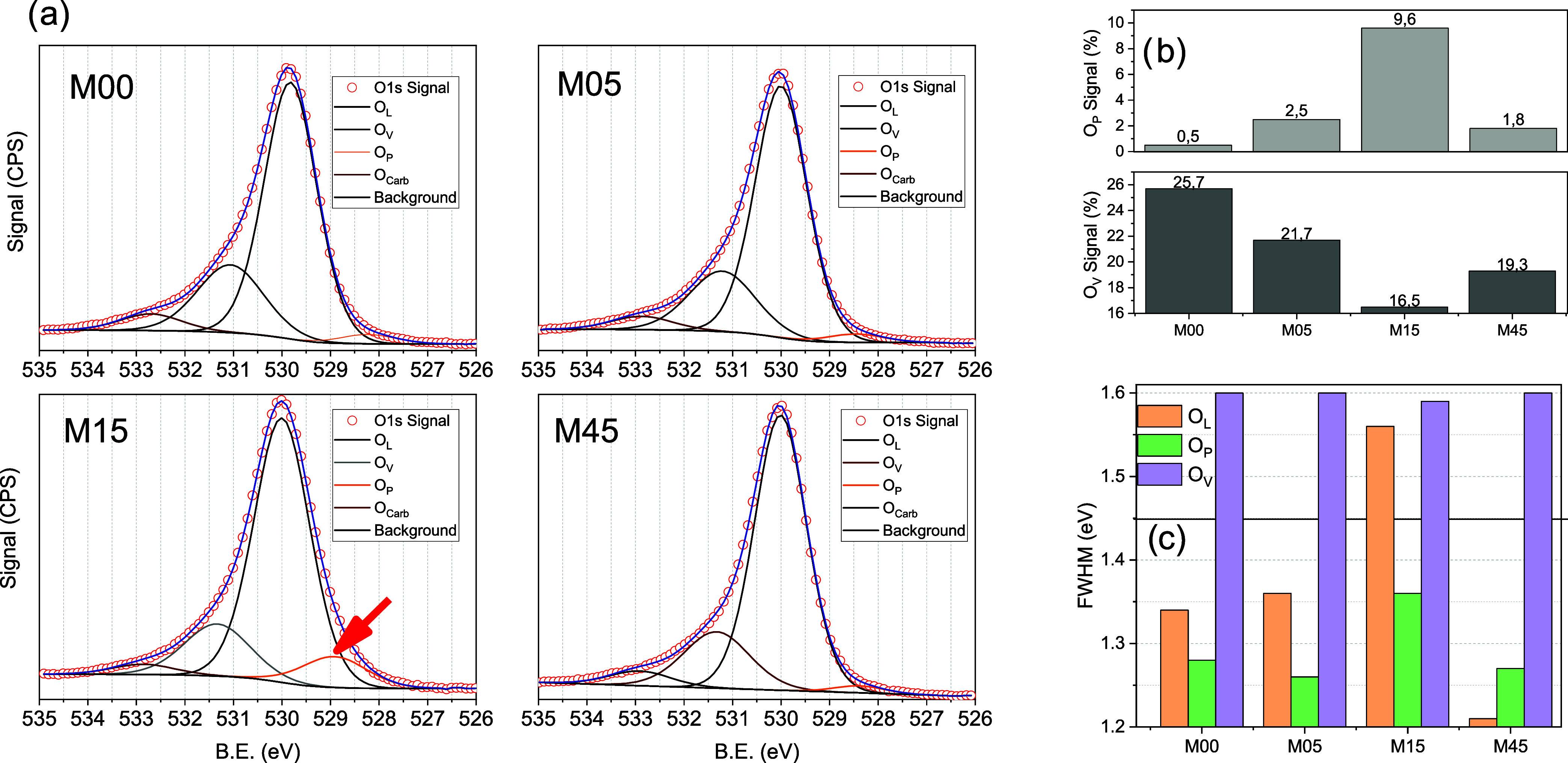
(a) High-resolution O 1s spectra collected for the samples under study. (b) Evolution of the O_V_ and O_P_ intensities and (c) O 1s components FWHM.

**Figure 8:**
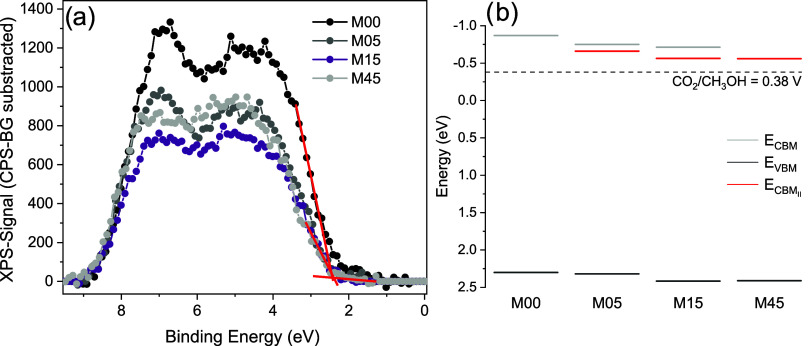
(a) XPS valence band spectra with corrected background. (b) Valence and conduction band potentials obtained from VB-XPS and DRS results. In red are marked the electronic states corresponding to the high-pressure polymorph.

The Ti 2p high-resolution spectra (Figure 6a) exhibit a systematic shift of both spin–orbit components toward higher binding energy as milling progresses. For Ti 2p_3/2_, the maximum moves from 458.48 eV in M00 to 459.12 eV in M45, while Ti 2p_1/2_ shifts from 464.16 to 464.79 eV over the same interval. These shifts point to modifications of the near-surface chemical environment, likely related to the introduction of defects, strain and changes in local coordination under mechanical milling. The spin–orbit splitting of 4.71 eV is consistent with Ti^4+^ as the predominant oxidation state.^57^ The absence of additional components at 456.8–457.8 eV and 462.5–463.5 eV indicates that no distinct Ti^3+^ contribution is detected at the surface within the sensitivity of XPS,^58^ which is in line with the known tendency of surface Ti^3+^ to be readily oxidized by O_2_ in air.^59^

Closer inspection of the Ti 2p_3/2_ line reveals an asymmetry on the low binding energy side for M15. Although similar features have been previously attributed to low concentrations of surface Ti^3+^,^60^ EPR measurements on the present samples do not support the formation of surface paramagnetic Ti^3+^ species, so this asymmetry is more plausibly associated with an increased dispersion of surface Titanium coordination environments. This interpretation is corroborated by the evolution of the full width at half-maximum (FWHM) of the Ti 2p_3/2_ peak: M00 displays a FWHM of 1.15 eV, which remains unchanged in M05, then widens to 1.30 eV in M15 (where TiO_2_–II reaches 28% at the surface), indicating increased disorder and/or a broader distribution of surface Ti sites. After 45 min of milling, the FWHM decreases to 1.21 eV, and the low binding energy tail is reduced, suggesting partial structural reorganization of the surface when TiO_2_–II becomes the dominant surface phase (60%).

The O 1s region (Figure 7) was fitted using four components associated with different oxygen species. The main peak, O_L_, corresponds to lattice oxygen in TiO_2_; O_V_ represents surface −OH groups; O_C_ is attributed to adsorbed carbonates or carbonyl species; and O_P_ is a less common low binding energy contribution without a straightforward conventional assignment. In M00, O_L_ is centered at 529.8 eV and accounts for 68.4% of the total O 1s intensity. As milling progresses, this component shifts to 530.0 eV, while its relative contribution increases up to 74.2% in M45. The O_L_/Ti ratio, reported in Table S.1, evolves from 1.9 (M00) to 1.8 for M05, M15 and M45, indicating that, although the absolute O_TOT_/Ti ratio decreases, the lattice oxygen contribution relative to Ti becomes slightly more uniform in the milled samples. The FWHM of O_L_ also shows a marked increase for M15 (1.56 eV), compared with M00 (1.34 eV), M05 (1.36 eV) and M45 (1.21 eV), pointing to a more strained and structurally heterogeneous surface in this intermediate milling stage.

The O_V_ component, located at 531.1–531.3 eV, decreases from 23.3% in M00 to 21.7% in M05 and reaches a minimum of 16.5% in M15, before rising again to 19.3% in M45. This trend indicates a reduction of surface hydroxyl groups from M00 to M15, with the lowest −OH coverage for the M15 sample and a partial rehydroxylation or reorganization at longer milling times. These OH signals has been commonly associated with surface hydroxyls or oxygen species adsorbed at oxygen vacancies (531–532 eV).^61^

The O_P_ peak, centered at 528.2–528.5 eV, remains at very low intensity (<2%) in M05 and M45, but becomes markedly more intense in M15, where it reaches ∼10% of the total O 1s signal (Figure 7b, top panel). Its FWHM also increases for M15 (1.36(1) eV vs 1.26(2) eV in the other compositions), consistent with a distribution of distinct local environments for these oxygen atoms. The binding energy of O_P_ is significantly lower than typical values reported for lattice oxygen in stoichiometric TiO_2_ (≈530.0 eV), Ti_2_O_3_ (530.8–531.2 eV) or TiO (531.0–531.5 eV),^62,57^ and also lower than those associated with surface hydroxyls or oxygen species adsorbed at oxygen vacancies (531–532 eV).^61^ Although initial chemisorption of O_2_ on metallic Ti can produce O 1s signals around 530–531 eV before full oxide formation,^63^ there is no evidence of metallic Ti in our samples. The unusually low binding energy of O_P_ therefore indicates a highly electron-rich oxygen environment. Given that M15 displays a high density of surface anatase/TiO_2_–II interfaces, it is reasonable to attribute these O_P_ species to oxygen atoms located in or near these strained interfacial regions, where local electronic density is enhanced. The pronounced negative shift of the O_P_ binding energy suggests a substantial increase of local electron density, rendering these sites strongly Lewis basic. Such electron-rich oxygens are expected to act as highly effective chemisorption and activation sites for CO_2_ (a Lewis acid), facilitating its adsorption and electronic activation and thus making it more susceptible to photoreduction.

Additional insight into the surface electronic structure is obtained from VB-XPS (Figure 8a). In the 0–10 eV region, pristine anatase (M00) exhibits the characteristic two main features assigned to O 2pσ (bonding) and O 2pπ (nonbonding) states,^64^ with well-resolved maxima and a sharp leading edge, consistent with a highly ordered surface electronic structure. After 15 min of milling, when the surface of M15 contains TiO_2_–II agglomerates of 20–30 nm on highly strained anatase grains, the valence-band spectra show a subtle shift of the overall envelope toward higher binding energy, a redistribution of the relative intensities of the O 2pσ and 2pπ features, and a less steep onset at the valence band edge. The separation between the valence band maximum (VBM) and the Fermi level (E_F_), extracted by linear extrapolation of the leading edge, increases from 2.30 eV in M00 to 2.42 eV in M15, with M05 and M45 yielding intermediate values of 2.32 and 2.41 eV, respectively.^65^ Since VB-XPS reports the VBM relative to E_F_, this increase implies that the near-surface levels become more n-type-like in energy alignment (i.e., the VBM moves farther from E_F_) as milling proceeds, particularly for the TiO_2_–II-rich samples M15 and M45. Within the limits of VB-XPS, this shift can originate from a change in E_F_ position and/or from surface band bending; the present data do not allow these contributions to be disentangled. Nonetheless, the consistent enlargement of the VBM-E_F_ separation combined with the line shape evolution (σ/π intensity redistribution and reduced onset slope) evidence a modified near-surface density of states in M15 and M45 in which TiO_2_–II contributions become significant.

By combining the VB-XPS analysis with the band gap values obtained from DRS, it is possible to estimate both VBM and conduction band minimum (CBM) positions with respect to E_F_ (Figure 8b). The VBM values derived in this way are 2.30, 2.32, 2.42, and 2.41 eV for M00, M05, M15 and M45, respectively, in agreement with the direct VB-XPS extrapolations. The corresponding CBM energies are −0.87 eV (M00), −0.75 eV (M05), −0.71 eV (M15) and −0.56 eV (M45). These CBM positions place all samples at sufficiently negative potentials to drive methanol production from CO_2_ (*E*_CO_2_/CH_3_OH_ = −0.38 eV), while the observed evolution reflects subtle but systematic changes in surface band alignment and electronic structure associated with the progressive incorporation of TiO_2_–II.

The combined XPS and EPR evidence indicates that high-energy ball milling induces a profound reorganization of vacancy-related electronic states in TiO_2_. In pristine anatase (M00), EPR detects isolated bulk Ti^3+^ centers associated with oxygen vacancies, whereas XPS does not resolve distinct Ti^3+^ components because surface Ti^3+^ is readily oxidized and subsurface species remain below the detection threshold, consistent with the established behavior of lightly reduced TiO_2_ surfaces.^62^ Upon milling, and particularly at the intermediate stage M15, the bulk paramagnetic Ti^3+^ signal decreases sharply, yet XPS continues to show no discrete Ti^3+^ signatures. This apparent divergence between techniques points not to the elimination of vacancy-derived electrons, but to a change in their electronic configuration under the intense strain fields, lattice distortions, and dense anatase/TiO_2_–II interfacial network generated by HEBM. The concurrent shift in the valence-band maximum and the modification of the valence-band line shape observed by VB-XPS support this redistribution of electronic density near the surface.

Theoretical and experimental studies provide a clear framework for interpreting this behavior. Liu et al. showed that oxygen vacancies in TiO_2_ may yield Ti^3+^–V_O_ complexes or donor states whose paramagnetic visibility depends on the degree of structural relaxation and coordination, allowing vacancy-derived electrons to adopt nonparamagnetic configurations.^69^ Di Valentin and Selloni demonstrated that lattice strain, asymmetric relaxation, and low-symmetry environments can drive vacancy electrons into singlet or extended states that are EPR-silent and do not appear as discrete Ti^3+^ features in XPS, despite remaining electronically active.^67^ Pan et al. further emphasized that defect-rich or strained TiO_2_ surfaces or interfaces stabilize a hierarchy of localized and extended donor states whose spectroscopic detectability varies with structural disorder.^68^ In parallel, Serpone et al. reported that deep traps and nonradiative centers in TiO_2_ can host electrons without producing isolated Ti^3+^ paramagnetic signatures.^66^ Collectively, these studies show that the highly distorted, interface-rich microstructure of M15 provides an environment where vacancy-derived electrons can reorganize into extended or paired configurations that are invisible to EPR and not resolved by XPS, yet significantly modify the near-surface electronic structure.

A further implication of this defect reorganization is reflected in the pronounced asymmetry of the Ti 2p_3/2_ envelope and in the emergence of the unusually low-binding-energy O_P_ component in the O 1s spectra, both maximized in M15. The Ti 2p asymmetry is consistent with an expanded distribution of Ti coordination environments arising from vacancy-induced distortions, low-symmetry bonding motifs, and the dense anatase/TiO_2_–II interfacial network generated by milling. Diebold has noted that oxygen-deficient or undercoordinated Ti sites produce broadened and asymmetric core-level responses,^62^ while Pan and Liu demonstrated that nonequivalent vacancy relaxations and deep-donor states enhance the heterogeneity of Ti–O electronic environments, leading to the type of tailing observed here.^66,68^ The sharp increase of the O_P_ component at 528.9 eV in M15 further supports the presence of electron-rich oxygen sites. According to DFT analyses by Di Valentin and Selloni, strained oxygen-deficient regions and interfacial bonding geometries can stabilize highly reduced O species with negative chemical shifts,^67^ while experimental studies summarized by Serpone show that deep-trap electron populations may reside in such centers without producing discrete Ti^3+^ signatures.^69^ The co-occurrence of Ti 2p asymmetry and a strong O_P_ contribution therefore reflects the formation of a highly distorted, electronically enriched near-surface environment characteristic of vacancy-rich and interface-dominated TiO_2_ under extreme mechanical strain.

### 3.4 Specific Surface Area and CO_2_-TPD Experiments

CO_2_-TPD profiles for M00 and M15 (Figure S.10) exhibit multiple desorption events between 130 and 500 °C, corresponding to distinct CO_2_-derived species. Deconvolution of the curves (Table S.2) identifies contributions from physisorbed CO_2_ (75–180 °C), bicarbonates (HCO_3_^–^, 180–380 °C), and bidentate carbonates (CO_3_^2–^, 380–570 °C), in agreement with previous assignments.^70^ Integration of the desorption profiles reveals that M15 releases nearly four times more CO_2_ than M00, demonstrating that milling generates a substantially larger population of adsorption sites. Notably, the high temperature region (>400 °C) becomes significantly more intense in M15, indicating the formation of stronger chemisorption centers.

These observations correlate with the XPS results: the broadened and asymmetric Ti 2p envelope and the emergence of the electron-rich O_P_ component in the O 1s spectra for M15 point to a distribution of defect-stabilized oxygen environments with increased local electron density. Such sites behave as stronger Lewis bases and favor the stabilization of tightly bound carbonate species that desorb only at elevated temperatures. Thus, HEBM does not merely increase the number of accessible surface sites; it alters their chemical nature by generating strained coordination environments, defect-derived electronic states, and anatase/TiO_2_–II interfacial regions capable of strengthening CO_2_ chemisorption.^30^

The evolution of CO_2_ adsorption must also be considered in the context of the specific surface area. BET analysis reveals a modest but systematic increase from 8.1 m^2^ g^–1^ (M00) to 11.3 m^2^ g^–1^ (M15) and 14.1 m^2^ g^–1^ (M45). Although this rise reflects crystallite-size reduction and increased surface heterogeneity, the magnitude of the enhancement in CO_2_ uptake, particularly in the strongly chemisorbed region, cannot be explained by surface area effects alone. Instead, it highlights the dominant influence of HEBM-induced defect chemistry and interfacial electronic structure on the binding strength and distribution of CO_2_ adsorption sites.

### 3.5 Photocatalysis and General Discussion

The photocatalytic behavior of the milled TiO_2_ samples exhibits two sharply contrasting trends depending on whether the reaction proceeds through an oxidative pathway (methyl orange degradation) or a reductive one (CO_2_ photoreduction). These opposite responses reflect the profound restructuring of the defect landscape, surface chemistry, and interfacial electronic structure induced by high-energy ball milling (HEBM), as established by XRPD, TEM, EPR, DRS, XPS, and CO_2_-TPD analyses.

Pristine anatase (M00) presents the highest efficiency for methyl orange (MO) degradation (92% discoloration after one hour, Figure 9a). This activity consists of its well-ordered bulk and surface, together with oxygen-related paramagnetic defects, surface Ti^3+^ centers, and oxygen vacancies, identified by EPR and supported by TEM and XRPD (Figures 1–5). These species promote charge separation and ^•^OH radical generation, the dominant oxidative pathway in MO degradation.^71^ After only 5 min of milling (M05), the MO degradation efficiency drops to 67%. At this stage, XRPD and TEM detect the onset of surface TiO_2_–II nucleation and an increase in the anatase crystallite boundary concentration, while EPR shows the disappearance of surface Ti^3+^ states. Both effects disrupt the electronic homogeneity and oxidative capability of anatase. In M15 (28 wt % TiO_2_–II), efficiency decreases to 51%, and at M45 (60 wt % TiO_2_–II), it falls to 22%. The progressive accumulation of strained TiO_2_–II domains, accompanied by increasing microstrain and the proliferation of extended defects (grain boundaries, dislocations, and anatase/TiO2-II interfaces), accelerates electron–hole recombination and suppresses hydroxyl radical formation.^72^ These observations align with XPS results showing broadened and heterogeneous Ti–O coordination environments. Stability tests (Figure S.11) confirm that the performance of M15 remains unchanged upon reuse, indicating that the reduced MO activity is intrinsic rather than caused by catalyst deactivation.

**Figure 9:**
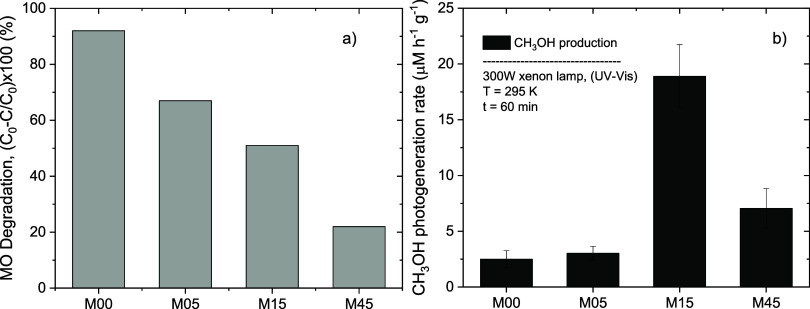
(a) MO degradation and (b) methanol photogeneration rate from CO_2_ reduction under UV–vis irradiation and ambient conditions.

In contrast, the CO_2_ photoreduction pathway displays a markedly different evolution across the milling series (Figure 9b). Methanol formation increases slightly from 2.5 μmol L^–1^ h^–1^ g^–1^ (M00) to 3.0 μmol L^–1^ h^–1^ g^–1^ (M05), changes that may arise from increased surface area and moderate defect activation.^73^ A dramatic enhancement occurs in M15, reaching ∼19 μmol L^–1^ h^–1^ g^–1^, an 8-fold increase compared to pristine anatase. This peak contrasts strongly with the suppressed MO degradation in the same sample, indicating that the reductive pathway exploits a completely different set of active sites and surface electronic states.

TPD-CO_2_ measurements (Figure S.10 and Table S.2) reveal that M15 exhibits a 4-fold increase in total CO_2_ desorption relative to M00, with a pronounced enhancement of high-temperature desorption events (>400 °C). These features indicate the formation of new strongly basic chemisorption sites.^63^ XPS corroborates this scenario: M15 displays an unusually low O 1s binding-energy component (O_P_ = 528.9 eV, ∼10% of the total intensity), far below the values reported for lattice oxygen in TiO_2_, Ti_2_O_3_, or TiO,^62,57^ or for vacancy-associated hydroxyl species (531–532 eV).^73^ This O_P_ contribution indicates highly electron-rich oxygen environments located at strained, nonstoichiometric interfacial regions. DFT studies by Di Valentin and Selloni show that such strained or oxygen-deficient environments can stabilize deeply reduced oxygen species with strong negative core-level shifts,^67^ while Serpone et al. demonstrated that deep electron-trap centers may not generate discrete Ti^**3+**^ features detectable by EPR or XPS.^69^ The coincidence of O_P_ growth with the strong increase in high-temperature CO_2_ adsorption (TPD) identifies these electron-rich oxygens as effective Lewis-basic sites for CO_2_ polarization and activation.

These basic oxygen sites coexist with strained, undercoordinated Ti atoms produced by high microstrain and by the ∼6% density mismatch between anatase (3.78 g cm^–3^) and TiO_2_–II (4.14 g cm^–3^). Their close spatial proximity, combined with geometric frustration preventing acid–base neutralization, produces an arrangement reminiscent of surface frustrated Lewis pairs (FLPs).^71,74,75^ FLPs are known to cooperatively activate CO_2_, polarizing the molecule at the basic site while stabilizing electron transfer at the acidic site. Such FLP-like behavior provides a plausible framework to explain the exceptional CO_2_ photoreduction efficiency observed in M15 and the synergy between anatase and TiO_2_–II contributions.

Additional structural justification for the heightened reactivity of these interfaces comes from the work of Zhao et al.,^24^ who showed that the (112)­A/(100)­II anatase/TiO_2_–II junction undergoes a modeled ∼6% area contraction and Ti–O bond-length variations up to 0.07 Å, generating a strongly strained region whose bonding geometry is intermediate between the two parent structures. These distortions produce both electron-rich O atoms and acid-like undercoordinated Ti sites, exactly the spectroscopic fingerprints observed in M15 through the O_P_ peak and the Ti 2p asymmetry. As milling proceeds to 45 min, TiO_2_–II domains grow and coalesce, reducing the density of such strained interfacial junctions. This structural evolution aligns with the reduced O_P_ intensity, narrower Ti 2p envelopes, partial strain relaxation (XPS), and the concomitant decrease in methanol production observed for M45.

The disappearance of paramagnetic Ti^3+^ signals in EPR across the series does not contradict these findings. Instead, it reflects the redistribution of vacancy-derived electrons from isolated Ti^3+^ centers (0D defects) into extended states stabilized at interfaces, dislocations, and grain boundaries (1D and 2D defects). XRPD evidence a strong increase in microstrain, consistent with the proliferation of extended defects acting as sinks for vacancy electrons.^76^ Theoretical works by Liu, Di Valentin, Pan and Serpone^66–69^ predict that such electrons can reorganize into paired, singlet, or delocalized states that are EPR-silent and do not produce discrete Ti^3+^ signals in XPS, while remaining catalytically active. VB-XPS confirms a modified near-surface density of states in M15 and M45, consistent with the progressive incorporation of TiO_2_–II contributions and redistribution of vacancy-related electronic states. These reorganized electronic structures do not support MO oxidation, which depends on isolated vacancies and Ti^3+^, but do enhance CO_2_ activation and reduction efficiency.

At longer milling times (M45), the decline in methanol production (∼7 μmol L^–1^ h^–1^ g^–1^) can be attributed to (i) excessive structural distortion reducing efficient charge separation, (ii) the emergence of rutile nanocrystals acting as recombination centers at the very surface layers, and (iii) the overaccumulation of TiO_2_–II diminishing the density of strained anatase/TiO_2_–II junctions and altering surface chemistry unfavorably.^21,22,28–31^ This combination results in less favorable adsorption, weaker electronic activation of CO_2_, and poorer charge-carrier utilization.

Comparison with established photocatalysts in the literature highlights the exceptional performance of the mechanically engineered anatase/TiO_2_–II heterostructure. Akhter et al.^77^ reported methanol production rates of only ∼3.2 μmol L^–1^ h^–1^ g^–1^ for mesoporous TiO_2_ (190 m^2^ g^–1^), whereas the present M15 sample, produced without doping, templating, or cocatalyst addition, achieves 19 μmol L^–1^ h^–1^ g^–1^ under UV–vis illumination. A broader comparison of TiO_2_-based and metal-doped photocatalysts is presented in Table 3, confirming that mechanical activation and phase-boundary engineering alone provide a competitive and sustainable route for improving CO_2_ photoreduction activity.

**Table 3:** TiO_2_ Photocatalysts Applied for CO_2_ Reduction and Their Respective Major Products Reported in the Literature

semiconductor	experimental method	products synthesis	refs
mesoporous TiO_2_	300 W UV lamp, 50 mL/min flow rate, H_2_O/CO_2_ = 0.1	2.63 μmol g_cat_^–1^ h^–1^ CH_4_	^67^
		5.44 μmol g_cat_^–1^ h^–1^ CH_3_OH	
		6.88 μmol g_cat_^–1^ h^–1^ CO	
Brookite-Lys TiO_2_ nanoparticles	500 W UV–vis lamp, NaHCO_3_ 0.1 mol L^–1^ with CO_2_	0.26 μmol g_cat_^–1^ m^–2^ CH_3_OH	^78^
ultrathin TiO_2_ flakes	300 W UV–vis lamp, H_2_O with 0.5 mL/min flow rate CO_2_	1.90 μmol g_cat_^–1^ h^–1^ CHOO	^79^
TiO_2_ anatase particles	8 W Hg lamp, 0.2 mol L^–1^ NaOH saturated with CO_2_	0.5 μmol g_cat_^–1^ h^–1^ CH_3_OH	^80^
		3.9 μmol g_cat_^–1^ h^–1^ CH_4_	
anatase TiO_2_ nanocubes	300 W UV–vis lamp, 0.25 mL of 4.0 mol L^–1^ HCl + 0.12 g NaHCO_3_	4.56 μmol g_cat_^–1^ h^–1^ CH_3_OH	^81^
		1.48 μmol g_cat_^–1^ h^–1^ CH_4_	
TiO_2_ particles	500 W high-pressure UV–vis lamp, 0.08 mol L^–1^ NaHCO_3_	0.48 μmol g_cat_^–1^ h^–1^ CH_3_OH	^82^
TiO_2_ nanosheets	Two 18 W low-pressure Hg lamps, 2.0 mol L^–1^ NaOH saturated with CO_2_	0.204 μmol g_cat_^–1^ h^–1^ CH_4_	^83^
		0.106 μmol g_cat_^–1^ h^–1^ CO	
		0.18 μmol g_cat_^–1^ h^–1^ CH_3_OH	
		0.063 μmol g_cat_^–1^ h^–1^ CH_2_O	
Cu^0^–TiO_2_	Six 15 W UV–C lamps, 0.1 mol L^–1^ sodium oxalate solution at 15 °C saturated with CO_2_	1.025 μmol g_cat_^–1^ h^–1^ CH_4_	^84^
		4.318 μmol g_cat_^–1^ h^–1^ CO	
		7.353 μmol g_cat_^–1^ h^–1^ C_3_H_8_O	
		9.930 μmol g_cat_^–1^ h^–1^ CH_3_OH	
		13.37 μmol g_cat_^–1^ h^–1^ C_3_H_6_O	
		17.58 μmol g_cat_^–1^ h^–1^ CH_3_COOH	
black TiO_2_–coated Cu	500 W Xe lamp (0.220 W cm^–2^), 8 kPa of CO_2_ produced in situ by 0.5 mol L^–1^ H_2_SO_4_ solution with NaHCO_3_	8.083 μmol g_cat_^–1^ h^–1^ CO	^85^
		1.333 μmol g_cat_^–1^ h^–1^ CH_4_	
Ag–TiO_2_	300 W Xe lamp, H_2_O saturated with CO_2_	86.80 μmol g_cat_^–1^ h^–1^ CO	^86^
		9.400 μmol g_cat_^–1^ h^–1^ CH_4_	
Pd–TiO_2_	500 W Hg lamp, CO_2_ atmosphere, and heat	11.05 μmol g_cat_^–1^ h^–1^ CO	^87^
Pt–TiO_2_	150 W Xe lamp, H_2_O vapor saturated with CO_2_	∼190 ppm h^–1^ CH_4_	^88^
high-pressure TiO_2_–II polymorph	300 W UV–vis lamp, H_2_O saturated with CO_2_	19.0 μmol L^–1^ g_cat_^–1^ h^–1^ CH_3_OH	this work

Overall, the two photocatalytic reactions interrogate fundamentally different aspects of the evolving TiO_2_ defect landscape. MO degradation is maximized by ordered anatase and isolated Ti^3+^/oxygen-vacancy sites, which are progressively destroyed by milling. CO_2_ reduction, instead, is maximized when strained anatase/TiO_2_–II interfaces, electron-rich oxygen species, and interface-stabilized donor states (EPR-silent but electronically active) are most abundant, conditions that peak in M15. Mechanical energy therefore, reshapes the TiO_2_ defect architecture in a way that selectively suppresses oxidative pathways while enhancing reductive ones, establishing a unified framework for photocatalysis in mechanically activated oxides.

## 4 Conclusions

When TiO_2_ anatase is subjected to intense pressures and shear stresses during high-energy ball milling, significant structural modifications occur in both the bulk and surface regions, stabilizing TiO_2_–II high-pressure polymorph nanocrystallites. Surface and bulk properties of the samples were modified, resulting in a controllable polymorphic structure, which could be very useful for the design and preparation of new photocatalysts based on TiO_2_ using a green and simple synthetic technique. These changes enhance the photocatalytic CO_2_ reduction in aqueous media but impair the photooxidation of methyl orange. Notably, the methanol production rate from CO_2_ increases 8-fold for M15, a polymorphic arrangement revealing a complex interplay between the bulk and surface properties of TiO_2_ and its photocatalytic performance. Optimal activity is attained only when the surface concentration of the TiO_2_–II phase falls within a specific range (approximately 30%). The presence of basic oxygen species at the surface of M15 and its coexistence with titanium cations in diverse environments like strained anatase, TiO_2_–II nanocrystallites, and anatase/TiO_2_–II interfaces could be the key to the efficient activation and subsequent photoreduction of CO_2_. These findings demonstrate that enhancing CO_2_ photoreduction can be effectively achieved through high-energy ball milling of TiO_2_ under conditions that induce the TiO_2_-anatase to TiO_2_–II transition. This low-cost and environmentally friendly approach emerges as a viable strategy to improve the photocatalytic performance of TiO_2_ in CO_2_ conversion processes. Further improvements could be pursued by incorporating chemical agents during the milling process, depositing metals or metal oxides, or inducing topotactic H_2_ reduction approaches that offer promising pathways to tune the surface and maximize CO_2_ photoreduction without relying on noble metals.

## Supplementary Material



## Data Availability

Data are contained within the article and Supporting Information. Further inquiries can be directed to the corresponding author.

## Notes

Surface atomic composition, 300 W UV–vis Xe lamp light spectrum, XRD diffractograms and Rietveld refinement, TEM images, Tauc Plots, XPS spectra, CO_2_-TPD profiles and deconvolution results, and reusability tests of the M15 photocatalyst in the photodegradation of MO (PDF)
