# Supplementary material for: Surface Stabilization of High-Pressure TiO2 Polymorph via High-Energy Ball Milling: Boosting Noble-Metal-Free CO2 Photoreduction
Source: ACS Omega. 2026 Mar 13;11(11):17404–17. doi: 10.1021/acsomega.5c10398 (PMC13019392; doi:10.1021/acsomega.5c10398)
Supplement: Supplementary file 1 [file ao5c10398_si_001.pdf]

SUPPORTING INFORMATION

Surface Stabilization of High-Pressure TiO_2 polymorph via High-Energy Ball Milling: Boosting Noble-Metal-Free CO_2 Photoreduction

Abigail Mufari[a]; Thiago Capelupi[b], Martin Saleta[c, d, e], Eugenia Zelaya[e], Octavio Furlong[f], Maria Valnice Boldrin Zanoni[b], Luis Eduardo Cadús[a], Juliana Ferreira de Brito[b*] and Sebastián Larrégola[a*]

[a] *Instituto de Investigaciones en Tecnología Química (INTEQUI), UNSL - CONICET, Almirante Brown 1455, 5700, San Luis, Argentina.*
[b] *Universidade Estadual Paulista (UNESP), Instituto de Química, Araraquara, Brazil*
[c] *Instituto de Nanociencia y Nanotecnología (INN), CNEA-CONICET, Centro Atómico Bariloche, Av. Bustillo 9500, 8400 S. C. de Bariloche (RN), Argentina.*
[d] *Instituto Balseiro, Universidad Nacional de Cuyo and CNEA, Av. Bustillo 9500, 8400 S. C. de Bariloche (RN), Argentina.*
[e] *Centro Atómico Bariloche, Comisión Nacional de Energía Atómica Av. Bustillo 9500, 8400 S. C. de Bariloche (RN), Argentina*

*Corresponding author:

Juliana Ferreira de Brito - juliana.f.brito@unesp.br

Sebastián Larrégola - larregolasebastian@gmail.com

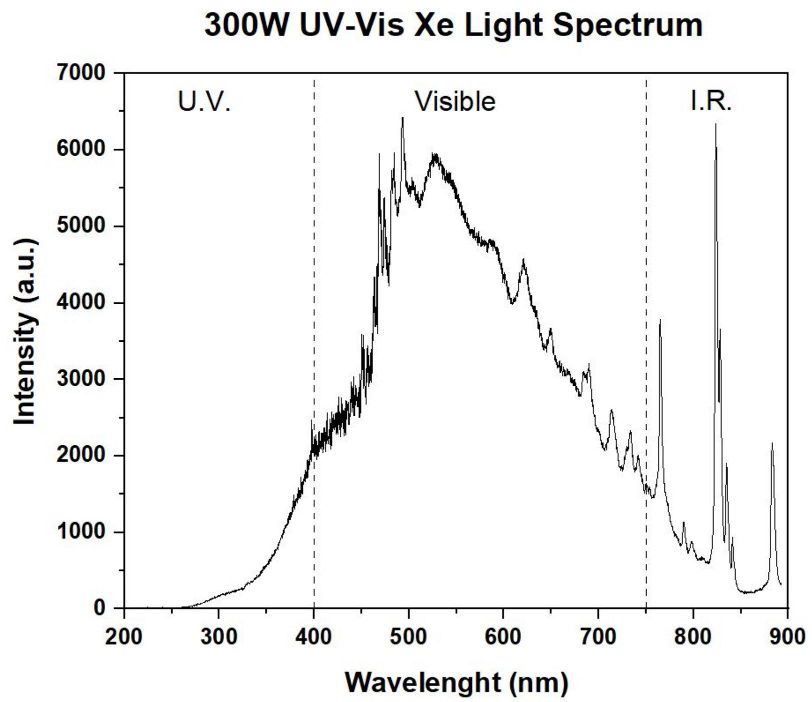

Figure S.1: 300 W UV-Vis Xe lamp light spectrum measured with Ocean Optics USB4000 Fiber Optic Spectrometer.

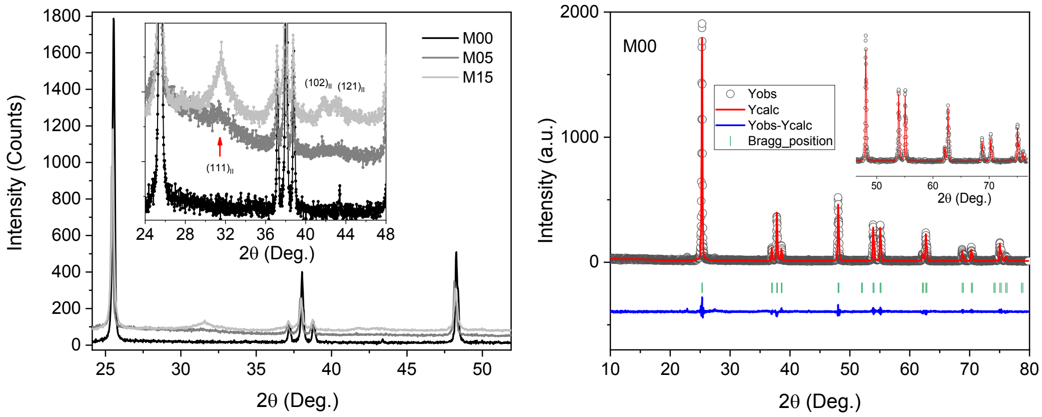

Figure S.2: a) Evolution of the X-Ray diffraction patterns collected for M00, M05 and M15 samples. Inset: Close up of the TiO_2-II reflections. b) Rietveld refinement plot of M00 sample.

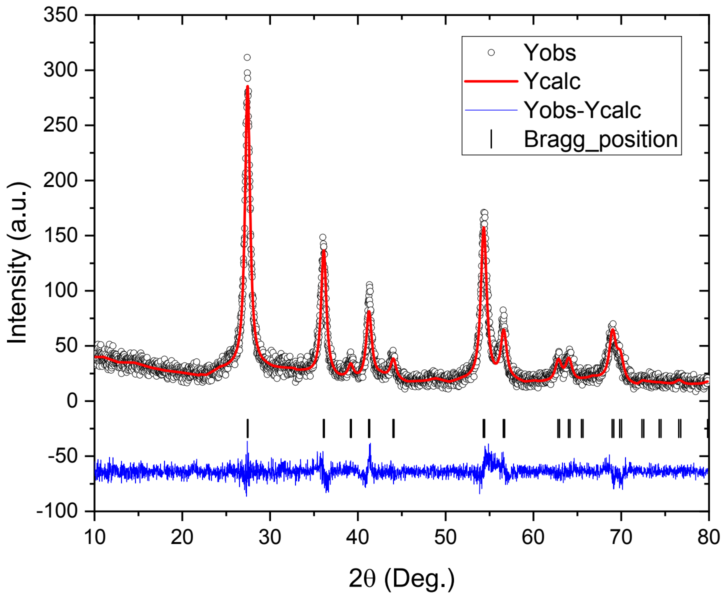

Figure S.3: Rietveld refinement plot for the simple milled for 360 min. Vertical ticks correspond to the rutile Bragg reflections.

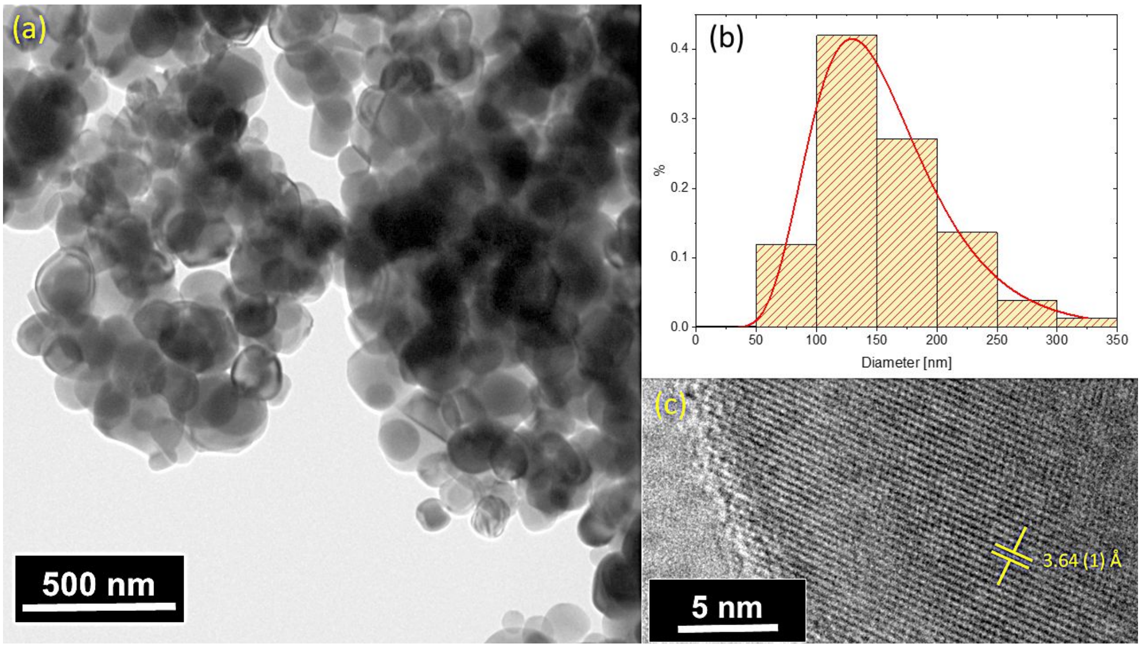

Figure S.4: TEM analysis of M00 sample. (a) Characteristic TEM image, (b) grain size distribution of the sample. The data were modeled with a log-normal distribution: <d> = 158 nm, σ = 0.35 nm. (c) HRTEM image of as made sample. The interplanar separation is 3.64 (1) Å, this value is close to the expected for the (100) plane of TiO_2-anatase.

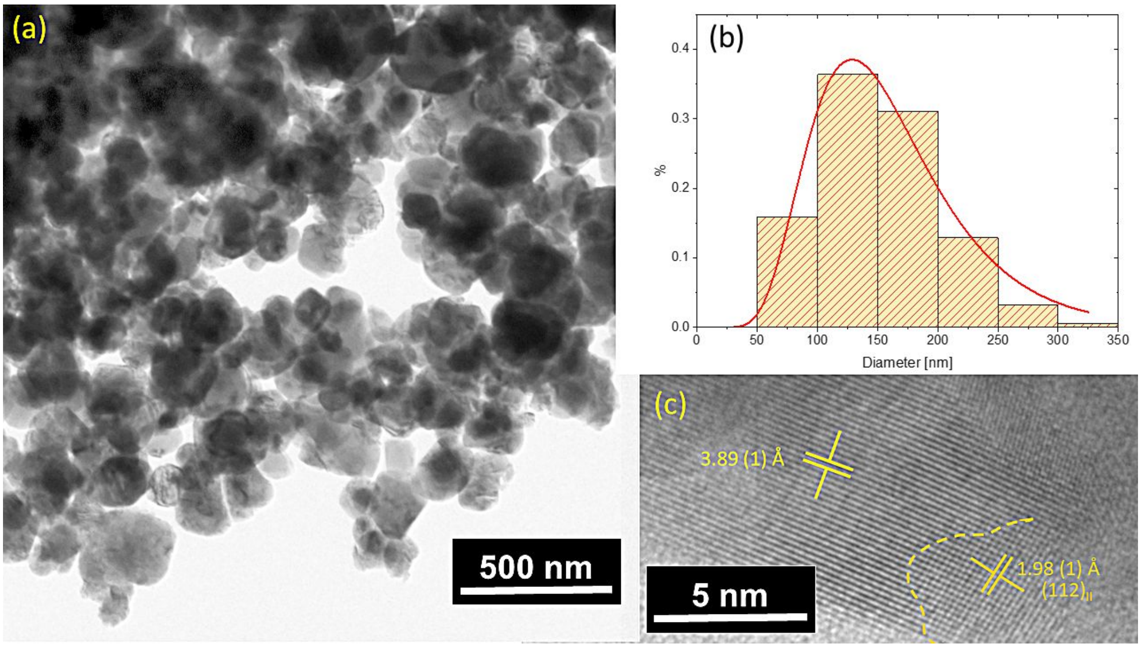

Figure S.5: TEM analysis of M-15 sample. (a) Characteristic TEM image, (b) grain size distribution of the sample. The data were modeled with a log-normal distribution: <d> = 158 nm, σ = 0.35 nm. (c) HRTEM image of as made sample. The interplanar separation is 3.89 (1) Å, was assigned to the anatase phase, while the 1.98 (1) Å distance corresponds to (112) of the TiO_2-II.

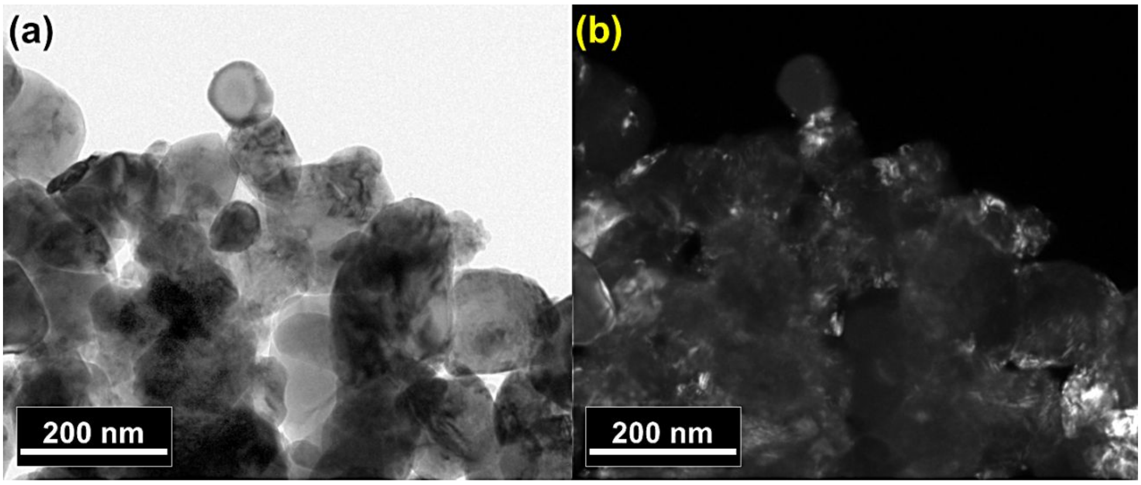

Figure S.6: (a) bright field and (b) dark field images. The dark-field images were constructed by positioning the objective aperture at the TiO_2-II diffraction ring. In these images, particles with sizes below 50 nm are evident.

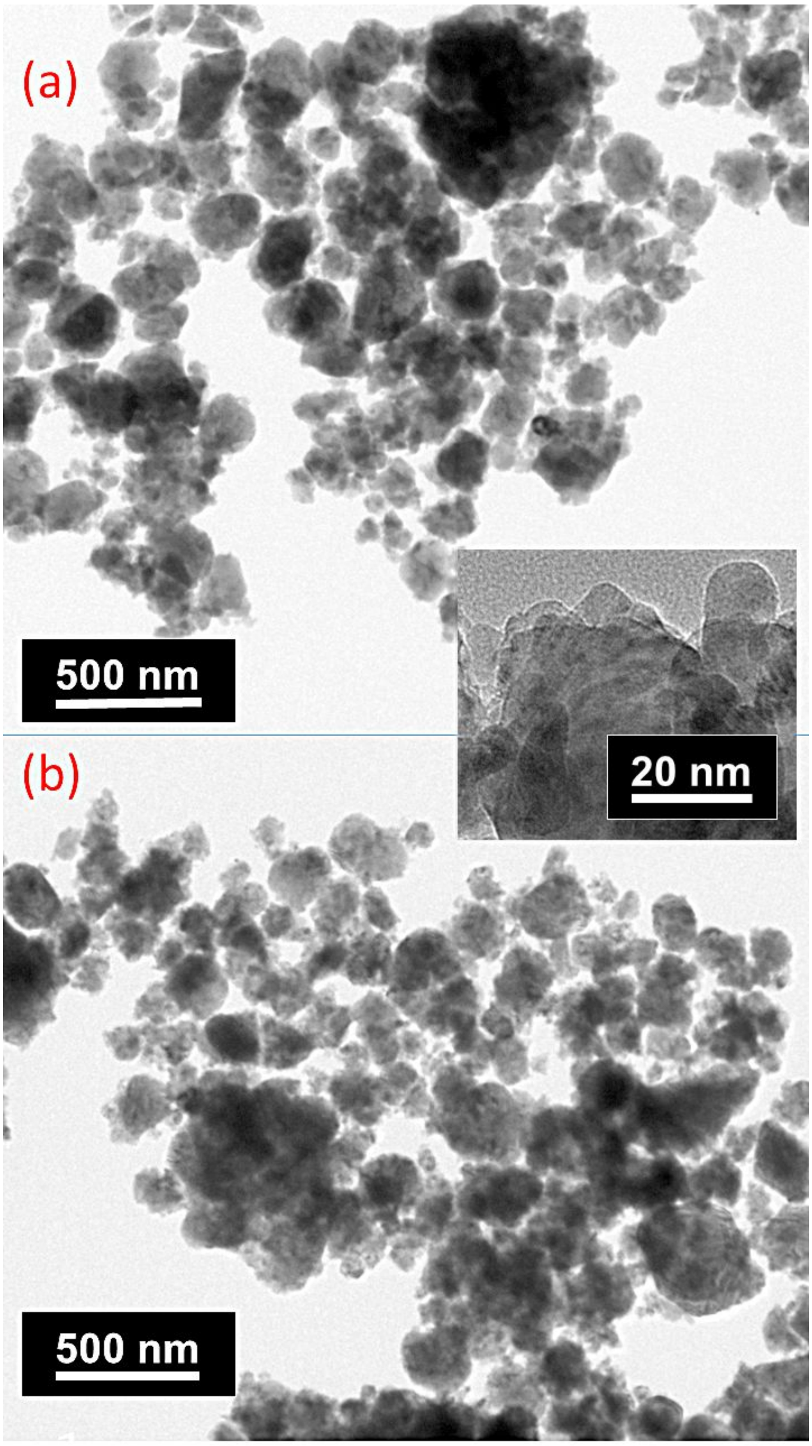

Figure S.7: a-b) Characteristic TEM image of the sample. Inset high magnification image where particles with a size smaller than 50 nm are observed.

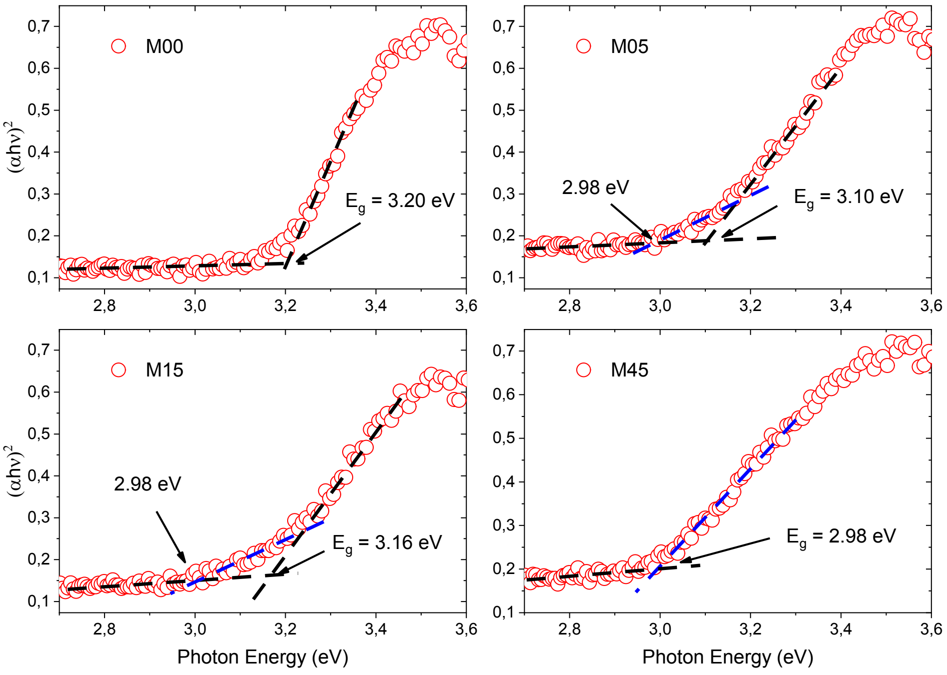

Figure S.8: Band gap determination from the Tauc Plots obtained for the samples under study. Linear extrapolations are shown as dashed lines.

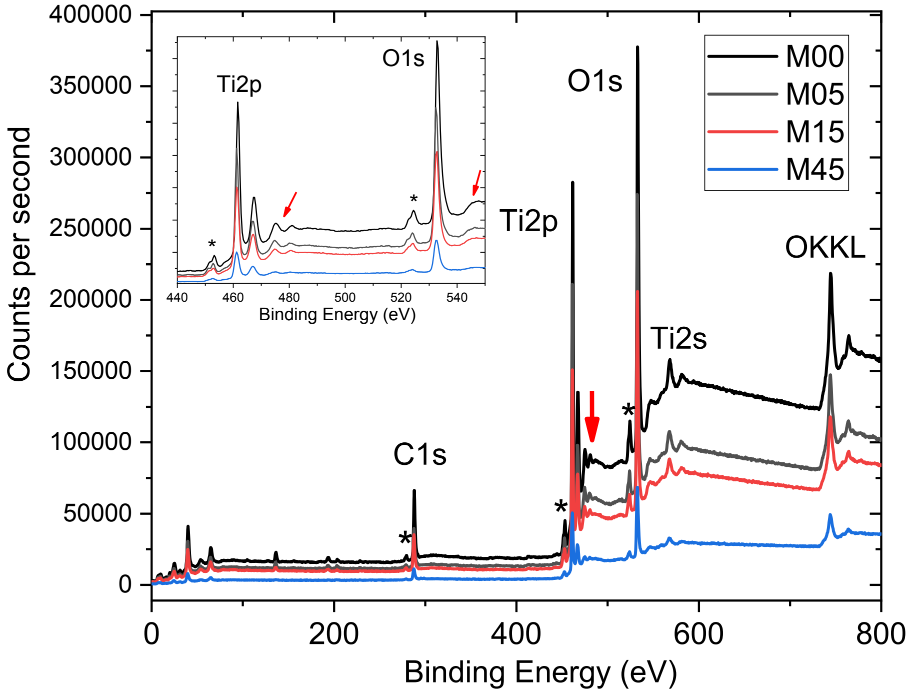

Figure S.9: Survey XPS spectra collected for all samples. The main components correspond to C, O, and Ti atoms. Plasmon loss satellites, marked with arrows, are observed for all core levels and are more intense in the Ti 2p region, appearing at energy separations of 13.5 and 19.4 eV from the main photoemission lines. Weak low-binding-energy replicas, marked with asterisks and separated by 8.4-10.2 eV from the main peaks, are attributed to Mg Kα_3/Kα_4 satellite radiation. Weak sharp features observed between

100 and 220 eV, invariant with milling, are attributed to instrumental effects of the X-ray source. Ref: *Briggs, D.; Seah, M. P. Practical Surface Analysis, Auger and X-ray Photoelectron Spectroscopy; 2nd ed.; John Wiley & Sons: Chichester, U.K., 1990. Wagner, C. D.; Naumkin, A. V.; Kraut-Vass, A.; Allison, J. W.; Powell, C. J.; Rumble, J. R. NIST X-ray Photoelectron Spectroscopy Database, Version 3.5; National Institute of Standards and Technology: Gaithersburg, MD, 2003.*

Table S.1: a) Surface atomic composition calculated from the survey spectra collected for the samples.

	M00	M05	M15	M45
**Ti (%)**	20.3	21.7	21.1	21.7
**O (%)**	54.7	55.3	53.3	53.4
**C (%)**	25.0	23	25.6	24.9
**O_{TOT}/Ti**	2.7	2.5	2.5	2.5
**O_L/Ti**	1.9	1.8	1.8	1.8
***O_V**	14.5	12.0	8.8	10.3
***O_P**	0.3	1.3	5.1	0.9
***O_C**	2.0	2.5	1.6	2.5

*Oxygen-components percent normalized to the total oxygen percent.

b) Oxygen 1s Core spectra deconvolution results.

O1s Signals												
	M00			M05			M15			M45		
**O 1s**	**B.E. (eV)**	**FWHM**	**$\%_{TOT}$**	**B.E. (eV)**	**FWHM**	**$\%_{TOT}$**	**B.E.(eV)**	**FWHM**	**$\%_{TOT}$**	**B.E.(eV)**	**FWHM**	**$\%_{TOT}$**
**O_L**	529.8	1.28	70.1	530.0	1.26	71.3	530.0	1.36	70.9	530.0	1.27	74.2
**O_V**	531.1	1.6	25.7	531.2	1.6	21.7	531.33	1.59	16.5	531.32	1.6	19.2
**O_P**	528.2	1.34	0.5	528.4	1.36	2.5	528.9	1.56	9.6	528.4	1.21	1.8
**O_C**	532.7	1.5	3.7	532.9	1.5	4.5	532.85	1.4	3.0	532.9	1.5	4.7

Table S.2: Deconvolution results obtained from the TPD-CO_2 experiments carried out for M00 and M15 samples. In bold are highlighted the chemisorption events: HCO_3^- (peaks 3 and 4) and CO_3^{2-} (peaks 5 and 6).

M00					M15				
**Peak**	**T_{max} (°C)**	**FWHM (°C)**	**Area**	**%**	**Peak**	**T_{max} (°C)**	**FWHM (°C)**	**Area**	**%**
1	146	22	1069	4.9	1	136	21	1960	4,3
2	168	34	2867	13.1	2	157	34	4404	9,7
3	200	55	4914	22.5	3	191	56	8453	18,5
4	284	99	9432	43.1	4	256	97	11473	25,2
5	391	96	3027	13.8	5	365	150	17407	38,2
6	490	57	566	2.6	6	506	88	1920	4,2

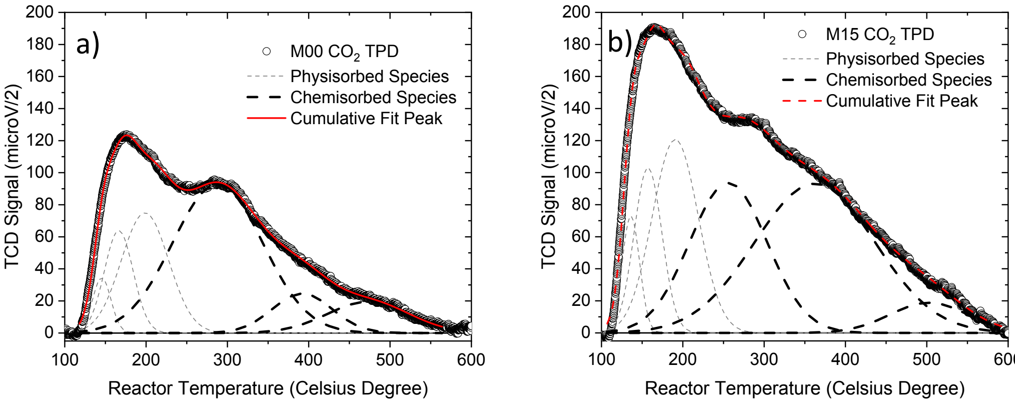

Figure S.10: CO_2-TPD profiles measured for M00 and M15 samples. Profile deconvolution as a function of temperature a) M00 and b) M15.

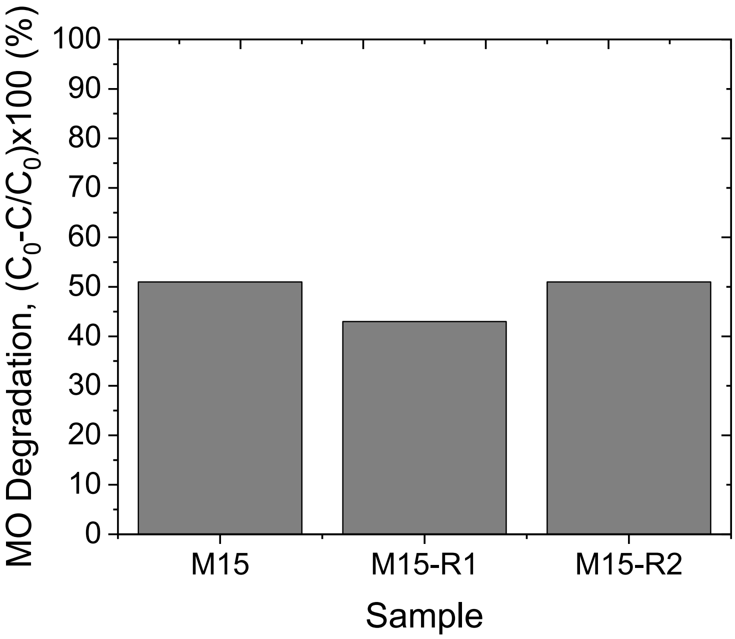

Figure S.11: Reusability tests of the M15 photocatalyst in the photodegradation of methyl orange (MO) during three consecutive 1-h runs: M15, M15-R1, and M15-R2.
